# In Vitro Differentiation of Myoblast Cell Lines on Spider Silk Scaffolds in a Rotating Bioreactor for Vascular Tissue Engineering

**DOI:** 10.3390/jpm12121986

**Published:** 2022-12-01

**Authors:** Doha Obed, Nadjib Dastagir, Christina Liebsch, Alperen S. Bingoel, Sarah Strauss, Peter M. Vogt, Khaled Dastagir

**Affiliations:** Department of Plastic, Aesthetic, Hand and Reconstructive Surgery, Hannover Medical School, 30625 Hannover, Germany

**Keywords:** myoblast, vascular tissue engineering, cell differentiation, spider silk, scaffolds, tissue engineering

## Abstract

Functional construction of tissue-engineered vessels as an alternative to autologous vascular grafts has been shown to be feasible, however the proliferation of seeded smooth-muscle cells remains a limiting factor. We employed a rotating bioreactor system to improve myoblast cell differentiation on a spider silk scaffold for tissue-engineered vessel construction. C2C12 myofibroblast cells were seeded on the surface of spider silk scaffold constructs and cultivated in a rotating bioreactor system with a continuous rotation speed (1 rpm). Cell function, cell growth and morphological structure and expression of biomarkers were analyzed using scanning electron microscopy, the LIVE/DEAD^®^ assay, Western blot and quantitative real-time PCR analyses. A dense myofibroblast cell sheet could be developed which resembled native blood vessel muscular tissue in morphological structure and in function. Bioreactor perfusion positively affected cell morphology, and increased cell viability and cell differentiation. The expression of desmin, MYF5 and MEF2D surged as an indication of myoblast differentiation. Cell-seeded scaffolds showed a tear-down at 18 N when strained at a set speed (20 mm min^−1^). Spider silk scaffolds appear to offer a reliable basis for engineered vascular constructs and rotating bioreactor cultivation may be considered an effective alternative to complex bioreactor setups to improve cell viability and biology.

## 1. Introduction

There remains a continuing increase in the use of vascular grafts in reconstructive procedures considering the increasing prevalence of microvascular free-flap tissue reconstructions. In light of this, autologous vein grafting remains an integral part in plastic and reconstructive surgery, as well as in cardiovascular surgery, the treatment of critical limb ischemia and arteriovenous fistula creation for hemodialysis access. However, autologous vein grafts, particularly the saphenous veins, may pose long-term limitations due to systemic hemodynamic and inflammatory changes, that can cause graft occlusion and failure in the long run [1]. Clinically available synthetic vascular substitutes similarly provide limited long-term function and do not allow for tissue growth and remodeling [2].

To overcome these challenges, several approaches to tissue-engineered blood vessels have been pursued to provide new alternative grafts, which may be customized and readily obtainable. Following numerous exploratory studies, the field of vascular tissue engineering has shown promising results. Various scaffold materials under different culture conditions and with various cell types have been put to the test [3,4,5]. A large amount of the reports in tissue engineering have relied on polymer-based hydrogels such as gelatin, agarose and fibrin. Still, mimicking the structural complexity and composition of blood vessels remains challenging as the examined biodegradable scaffolds have shown insufficient blood pressure resistance, impaired immunocompatibility and flawed responses to mechanical stimuli and temperature [2,6].

Contrastingly, spider silk has been shown to be a biodegradable and cytocompatible tissue generation material that may be used as a scaffold for vascular construction [7,8]. Apart from its significant biomechanical characteristics, e.g., its unusually high tensile strength and its thermal stability, there are a multitude of properties that render it a versatile biomaterial for application in vitro and in vivo: distinctive biomaterial morphologies, versatility in processing, ease of sterilization and chemical modifiability [7,9]. Recently, we have shown that in the generation of tissue-engineered vessels, spider silk allowed for an adequate scaffold for the in vitro cell culture of seeded smooth-muscle and endothelial cells [8]. For this purpose, a bioreactor perfusion system was setup to optimize cell proliferation and viability under dynamic and more physiologic culture conditions. Given the general understanding that dynamic culture conditions provide better outcomes for cell cultures in terms of cell function, cell differentiation and tissue structure organization [10], we opted to create a simpler bioreactor setup for the cell cultivation on a spider silk scaffold.

In the present analysis, we created a rotating bioreactor system with a continuous rotation speed, that would allow for an even mixture of the culture solution and oxygen and nutrient supply without the application of shear forces to the scaffold. The aim was to investigate C2C12 myoblast cell line differentiation in vitro on a spider silk scaffold for the development of a tissue-engineered vessel and to investigate the effects of this dynamic culture conditioning on cell function and growth as well as morphological structure and expression of biomarkers following a period of two weeks in a rotating bioreactor.

## 2. Methods

### 2.1. Silk Rearing, Scaffold Construction and Cell Seeding

**Figure 1:**
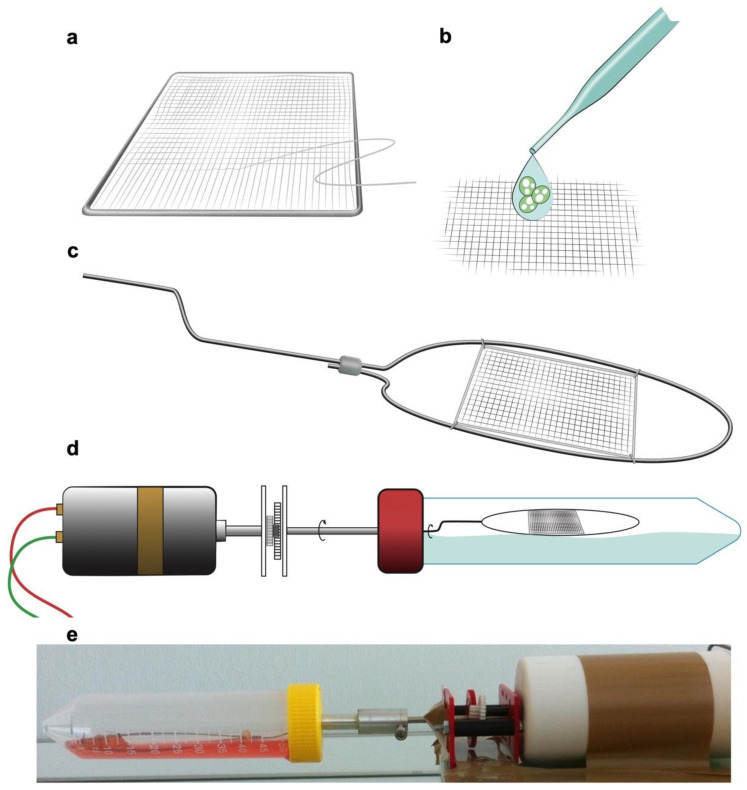
Scaffold and bioreactor construction. (**a**) Spider silk was manually sewn and woven in a parallel design on stainless-steel frames orthogonal to the run of the silk. (**b**) C2C12 myofibroblast cells were seeded and cultured on the spider silk scaffold. (**c**,**d**) Constructed and seeded scaffolds were mounted on a scaffold carrier which was stabilized by steel wires and loaded into a bioreactor with a continuously rotating scaffold carrier which was attached to an external motor, allowing repetitive immersion in the liquid with a speed of 1 rpm. (**e**) Picture of the rotating bioreactor setup for culturing of the spider silk constructs.

All procedures were conducted in accordance with the European Directives and the German Animal Welfare Law without approval since spiders as invertebrates do not necessitate allowances. No harm was done to the spiders in this study as silking presents a physiological process. Dragline silk harvesting as a standard procedure as well as animal keeping have previously been described in detail [11,12]. Briefly, spiders (*Nephila edulis*) were kept in humidified and warmed rooms and consumed crickets (*Acheta domesticus*) twice a week. Cobwebs were misted daily with tap water. For silk harvesting, adult female spiders were immobilized on polystyrene blocks with needle pins. Using sterile forceps, silk was drawn out of their major ampullate glands, reared on an extraction device and fixed on a motorized drum with a continuous speed of 4 cm·s^−1^. Strands were made out of two bundles and handwoven onto a stainless-steel frame (2.5 cm × 2.5 cm, 0.7 mm diameter) (Dentaurum, Ispringen, Germany) to create a mesh (Figure 1). The suturing was conducted with a needle in an orthogonal direction to the silk fibers’ course in a continuous method. The meshes were then autoclaved at 121 °C for 15 min, at 100% water vapor saturation and 2-bar pressure. Subsequently, the constructs were seeded with the C2C12 cells on one side of the scaffold.

### 2.2. Cell Culture

The usual approach to cell culture and isolation, as well as seeding, has previously been described in detail [8]. Myofibroblast cell lines C2C12 (ATCC, Manassas, VA, USA) were cultured in static culture conditions with Dulbecco’s Modified Eagle Medium (DMEM) High-Glucose, supplemented with 1% sodium pyruvate (100 mM), 1% penicillin/streptomycin (10,000 U/mL) and 10% fetal calf serum (FCS) (all Biochrom, Berlin, Germany). Prior detachment was conducted in 0.02% EDTA in phosphate-buffered saline w/o (PBS w/o), as well as 0.25% trypsin (wt./vol) (all Biochrom, Berlin, Germany).

### 2.3. Bioreactor Construction and Setup

To simulate physiologic in vitro conditions, the scaffolds were each placed in a bioreactor with a rotating scaffold carrier 48 h after cultivation. The spider silk meshes were mounted on a scaffold carrier which was stabilized by steel wires to avoid a collapse of the scaffold and was installed in a manner in which the carrier would not come into contact with the bioreactor walls. 

The rotating bioreactor was composed of a tubular polypropylene compartment with a filling volume of 50 mL, which was attached to an external motor with an adjustable speed (regulated to 1 rpm) (Figure 1). The carrier rotation allowed for constant mixing of the culture medium, leading to a levelled medium oxygenation. The scaffold, submersed in the culture medium, was rotated without application of shear stress.

The whole system was cultured in 25 mL of the aforementioned bioreactor medium. It was kept in a standard cell incubator under controlled conditions. After two weeks of bioreactor cultivation, the scaffold was detached from the bioreactor and arranged for further analyses. 

### 2.4. Live/Dead Assay 

We used Invitrogen’s Viability/Cytotoxicity Kit LIVE/DEAD^®^ assay (Darmstadt, Germany) for the cell viability assessment of C2C12 cells seeded on spider silk scaffolds after 48 h of cultivation. We adhered to the manufacturer’s directions. 

### 2.5. Scanning Electron Microscopy 

To report the ultrastructure of cells and morphologic changes on the spider silk scaffold before and after bioreactor incubation, we examined our samples by scanning electron microscopy (SEM). For this purpose, samples were fixed for 24 h following three weeks of cultivation using a sodium-cacodylate buffer (Merck, Darmstadt, Germany) and 2.5% glutaraldehyde (Polysciences, Warrington, PA, USA). Dehydration was performed by placement in increasing acetone dilutions. Upon drying using CPD030 (Bal-Tec, Balzers, Liechtenstein), the specimens were gold-coated utilizing an argon sputter (SEM Coating System, Polaron, East Grinstead, UK). The samples were then vacuum-secured and examined by SEM (SEM500, Philips, Germany). Images of the examination were taken according to the software and manual (Gebert & Preiss, 1998). 

### 2.6. Western Blot 

If not indicated otherwise, supplies were obtained from Sigma (Taufkirchen, Germany). The cell-seeded spider silk scaffolds and negative controls were lysed for blotting in 1% (vol/vol) Nonidet P-40, 0.5% (wt./vol) sodium deoxycholate, RIPA buffer (10 mM Tris (pH 8), 150 mM NaCl, 1 mM sodium orthovanadate, 1 mM phenylmethylsulfonyl fluoride (PMSF), 0.1% (wt./vol) sodium dodecyl sulfate (SDS), 4 µg/mL aprotinin). Commercially available samples of murine vessel tissue for positive controls (PC) were previously ground and lysed in PMSF, then added to RIPA. The concentration of protein was measured according to the Bradford method (Kruger, 1994). SDS polyacrylamide gel (PAGE) was laden with 25 μg of protein and subsequently shifted to Amersham Hybond ECL Nitrocellulose Membrane (GE, Buckinghamshire, UK). Membrane block was performed in 0.03% Tween-20 in PBS and 5% (wt./vol) skim milk, followed by incubation with the monoclonal murine anti-alpha-smooth-muscle-actin (α-SMA) (Chemicon, Millipore, Schwalbach, Germany) as the primary antibody. The donkey-anti-mouse (excitation/emission maxima: 679/696 nm) and donkey-anti-mouse (excitation/emission maxima: 778/795 nm) (LiCor, Bad Homburg, Germany) were used as secondary antibodies. Blot analysis was performed using an infrared fluorescence detector (Odyssey Infrared Scanner, LiCor, Bad Homburg, Germany). 

### 2.7. qPCR 

For total cellular RNA isolation from the scaffolds and negative controls, the NucleoSpin RNA II Kit (Macherey-Nagel, Dueren, Germany) was utilized. Murine vessel samples were used as positive controls and ground for RNA extractions utilizing TRIzol (Invitrogen, Carlsbad, CA, USA). Total concentrations of RNA were evaluated using spectrophotometry (NanoDrop, Wilmington, DE, USA). Then, 2% Tris-borate-EDTA (TBE) gel added with 0.5 μL of ethidium bromide (Sigma, Taufkirchen, Germany) were used for RNA quality confirmation, and 1 μg of total mRNA and iScript Reverse Transcription Supermix (Bio-Rad, Hercules, CA, USA) was used for reverse transcription. Amplification of diluted reverse-transcribed cDNA (5 μL) was performed using a 15 μL PCR assay volume, the target primer (Table 1), SsoFast EvaGreen Supermix (Bio-Rad, Hercules, CA, USA) and HPLC water. Gene expression was analyzed by qPCR, achieved with the Bio-Rad iCycler PCR machine. Analysis and normalization of data were carried out using qbasePlus (Biogazelle, Zwijnaarde, Belgium). Accordingly, the most firmly expressed genes among four normalization genes were identified by the GeNorm algorithm. qbasePlus enables utilization of numerous normalization genes, which is necessary for solid normalization. The expression levels of desmin, myogenic factor 5 (MYF5) and myocyte enhancer factor 2D (MEF2D) were normalized to those of TBP, RPL37 and b2-microglobulin (B2M), and stated as arbitrary gene expression units. The analyses were conducted in triplicates. 

### 2.8. Tensile Strength Test 

Ultimately, tissue-engineered vessels necessitate adequate mechanical strength to withstand the continuous high-pressure blood flow. To assess whether dynamic cultivation diminished the mechanical resistance, we conducted a tensile strength test. The mechanical properties of the cell-seeded spider silk scaffolds after dynamic bioreactor cultivation and the negative controls were determined by using a uniaxial tensile tester (Instron, Darmstadt, Germany). A section of 60 mm^2^ of both constructs was tested and strained at a set speed (20 mm min^−1^), yielding the stress–strain curves. Length variation (ΔL (mm)) and load (F (N)) were registered for both constructs (*n* = 3). 

### 2.9. Statistical Analysis 

All results are expressed as mean ± standard error of the mean. *n* indicates biological repeats of the conducted experiments, completed in triplicates. One-way or two-way ANOVA was used for comparisons of numerous experimental groups. When appropriate, we conducted Tukey’s multiple comparison test (95% CI).

## 3. Results

### 3.1. Scaffold Construction 

Spider silk arrangement could be achieved by manually sewing and weaving the silk strands into meshes measuring 10–100 μm. These meshes, as elastic scaffolds, allowed for cell layering and fixation. Under dry conditions, the width of the scaffolds measured, on average, 20 mm. Upon contact with a liquid medium, the scaffolds shrunk to approximately 74.3% of the preceding size, owing to the contractility of spider silk. In this study, a total of three scaffolds were created and analyzed. 

### 3.2. Cell Attachment and Viability 

**Figure 2:**
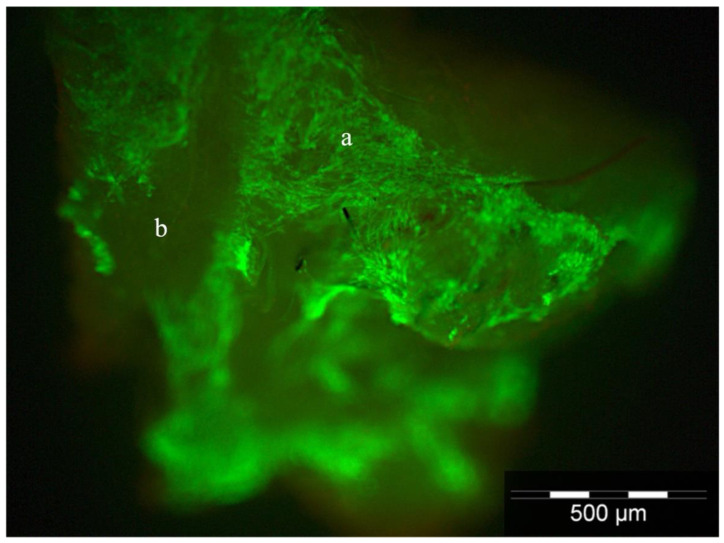
Visualization of cell viability and attachment of C2C12 myofibroblast cells (a) on the spider silk scaffold by LIVE/DEAD^®^ staining. (b) Marks the fibers of the auto-fluorescent spider silk.

The myofibroblast cells on the scaffolds were stained by LIVE/DEAD^®^ staining to visualize and evaluate attachment and cell viability. More than 95% of the cells could be discriminated from dead cells. The cell distribution could be demonstrated as a compact sheath configuration along the spider silk fibers. The scaffolds were not infiltrated by the cells (Figure 2). 

### 3.3. Scaffold Morphology and Cellular Properties 

**Figure 3:**
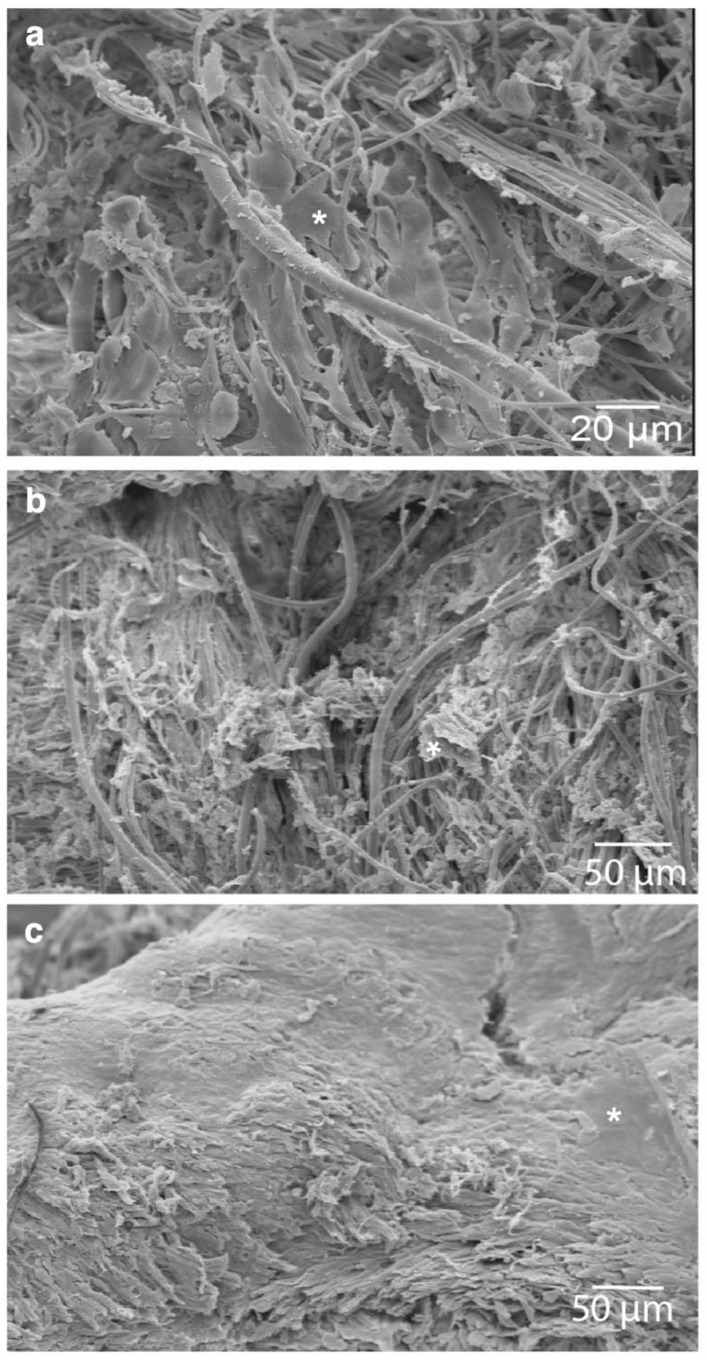
Ultrastructure and morphologic changes of C2C12 cells (*) on the spider silk scaffold visualized by scanning electron microscopy: (**a**) 3 h after cell seeding on the scaffold, (**b**) controls after 2 weeks without bioreactor cultivation and (**c**) 2 weeks after bioreactor cultivation.

To examine the ultrastructure of the cells and morphologic changes on the spider silk scaffold, the samples were examined by SEM. The cells on the scaffolds showed an orientation along the spider silk under static conditions. The adherent cells on the scaffolds altered their structural alignment and showed an increase in cell layer density (Figure 3). 

### 3.4. Western Blot 

**Figure 4:**
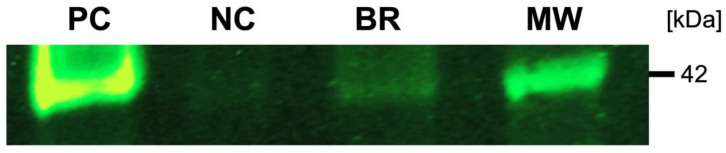
Marker protein analysis using Western blot. α-SMA cell-seeded scaffold cultured for two weeks in the presence of a bioreactor (BM) and in the absence of a bioreactor as a negative control (NG), murine blood vessels as a positive control (PC) and marker lane (MW).

Marker protein expression indicative of smooth-muscle cells was examined by Western blot. The results of the Western blot denoted the synthesis of explicit smooth-muscle cells’ protein markers. α-SMA could be identified in the scaffolds’ and positive controls’ protein fractions (Figure 4). 

### 3.5. qPCR 

**Figure 5:**
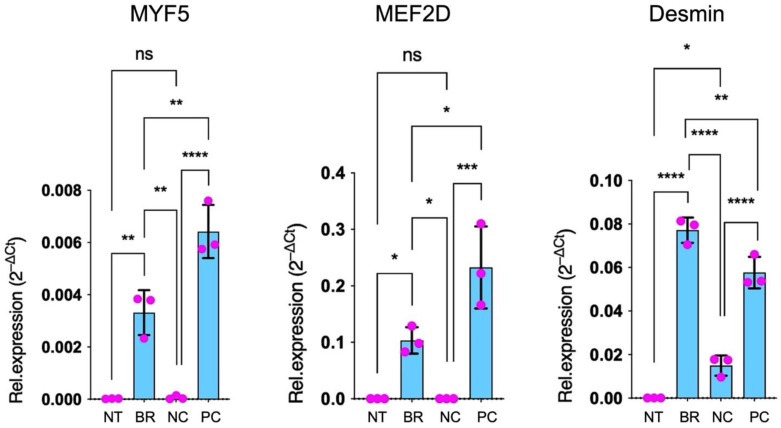
qPCR for the analysis of specific genetic marker expression (MYF5, MEF2D, desmin) for myoblast differentiation. Cell-seeded scaffolds were cultured for two weeks in the absence of a bioreactor as a negative control (NC) and in the bioreactor (BR), and murine blood vessels were utilized as a positive control (PC) in three independent experiments (error bars denote the standard error of the mean, ns = *p* > 0.05, * = *p* ≤ 0.05, ** = *p* ≤ 0.01, *** = *p* ≤ 0.001 and **** = *p* ≤ 0.0001).

Gene-specific (three) and normalization gene (four) primer pairs were devised (Table 1) for quantitative PCR assessment of specific genetic marker expression of vessel smooth-muscle cells. Expression levels of the examined genes were normalized to those of control samples and stated as arbitrary gene expression units. Desmin-, MYF5- and MEF2D-specific primer pairs were utilized to conclude whether the stimulated and cultured cells could undergo differentiation into active vascular cells. Differentiation for C2C12 cells was indicated by a surge in desmin, MYF5 and MEF2D expression in the scaffolds compared to negative controls. Accordingly, the PC were positive for these target genes, too. All examined target genes used for confirmation of active smooth-muscle cells showed higher expression in the scaffolds when compared to the negative controls (Figure 5). 

Desmin expression, marking a more advanced differentiation, was expressed with an increased intensity in the scaffolds from the rotating bioreactor compared to the PC. 

### 3.6. Tensile Strength Test

**Figure 6:**
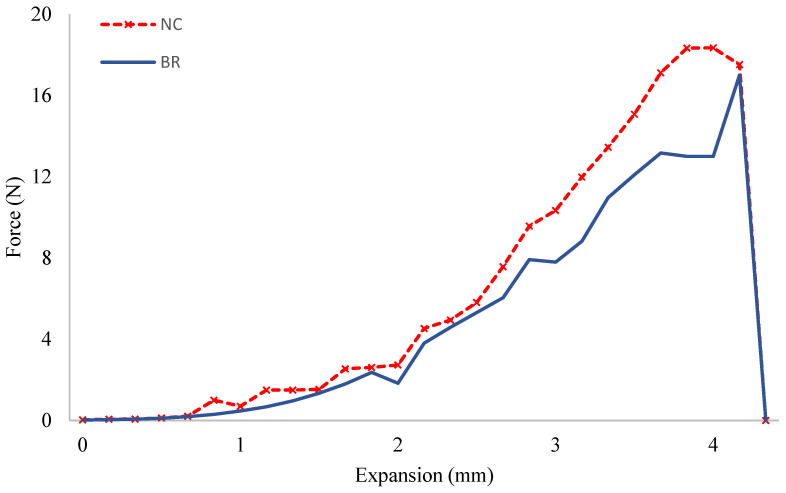
The mechanical properties of the cell-seeded scaffolds cultured for two weeks in the absence of a bioreactor as the negative control (NC) and in the bioreactor (BR) were tested by a tensile test. The breaking point was comparable for both constructs given a force of around 18 N.

The tensile test showed comparable results among the grafts undergoing dynamic cultivation and the negative controls. Regarding the elongation at break, our results indicated that the maximum force was found to be around 18 N for both constructs before the tear-down. The elongation at break demonstrated that negative controls endured more elongation before the breaking point compared to the dynamic grafts (3.83 vs. 3.81 mm). However, no statistically significant difference could be detected (Figure 6).

## 4. Discussion

For a broad spectrum of clinical indications, the need for tissue-engineered blood vessels to overcome the challenges that are posed by autologous vein grafts and synthetic vascular substitutes remains. To date, autologous grafts are marked by long-term limitations based on systemic hemodynamics and inflammatory changes, leading to graft occlusion and failure [13]. Similarly, synthetic vascular grafts are mostly nonviable and allogeneic products that are void of growth and remodeling capacities [14]. The clinical demand has become the drive for innovative approaches to therapeutic alternatives, pushing the advances in research of tissue-engineered vessels. The aim of this new approach to tissue regeneration is based on a concept in which biodegradable scaffolds are seeded with functional cells to create tissue structures. In recent years, considerable progress in the field of vascular tissue engineering has been made with demonstrations of clinical applicability in animal models and humans for cardiovascular applications [5,15,16,17,18,19]. 

For the elaboration of tissue-engineered vessels, a fitting scaffold for the promotion of vessel reconstruction is of utmost importance. The reconstruction outcome remains dependent on the various biomechanical characteristics and biochemical signals from the scaffold used [20,21]. Previous studies have underscored the significance of the scaffold’s biophysical assets, e.g., sturdiness, structure and porosity, for physiologic function and growth of the seeded cells [22,23]. 

Even though it could be demonstrated that the functional construction of tissue-engineered vessels seeded with endothelial and smooth-muscle cells was indeed feasible, the proliferation potential of the seeded sells has proven to be limited. Additionally, further reports have shown that the seeded cells, particularly the smooth-muscle cells, may have impaired function after cell expansion in vitro [24]. Smooth-muscle cells are crucial for maintaining graft integrity and stability and function as gatekeepers of vessel elasticity and radial compliance through their secretory capabilities [25]. Consequently, their quality and functionality are of paramount importance since they will influence all the subsequent outcomes. Previous studies have shown benefits in cell growth and cell functionality in tissue-engineered vessels when set in bioreactors with adequate mechanical environments [26,27]. Dynamic cultures have proven to be a necessity for in vitro generation of tissue-engineered vascular grafts [28,29], in contrast to static cultures, which have shown reduced nutrient and oxygen diffusion and are void of pulsatile stimuli, which are physiologic to vessel walls in vivo [10]. Perfusion bioreactors have proven to be effective for mechanical stimulation in various reports and we have previously shown their positive effects on cell viability and cell function of endothelial and smooth-muscle cells seeded on spider silk scaffold tissue-engineered vessels [8,30]. However, the complex setup may have posed a limitation for its broader clinical applicability. The present study’s objective was to deliver evidence and to optimize myofibroblast growth on a spider silk scaffold in a simpler rotating bioreactor model.

For this purpose, the seeded spider silk scaffolds where exposed to a rotating bioreactor drum without a significant application of mechanical stimulation to the constructs after two weeks of culture, and the cells were analyzed histologically and biochemically in comparison to scaffolds cultured under static conditions. Our results have shown to be consistent with preceding studies concerning the influence of a rotating bioreactor on cell density and cell function measured by target gene and protein analysis [31,32].

Our experimental setup induced significant alterations of cell morphology in the rotated and static constructs, which we attributed to the repetitive immersion of the cells in the fluid and the mimicking of minimal shear stress conditions. The changes in cell density and orientation, as well as overall morphology, have been demonstrated by SEM imaging (Figure 4). The creation of a dense sheet of C2C12 cells that extended across the matrix showed to be promising for implementation in tissue-engineered vascular grafts as a muscular layer. Multiple studies have elaborated on the reaction of smooth-muscle cells to mechanical stress and have highlighted our observed results regarding cell elongation towards the flow direction and cytoskeletal grouping [33,34]. 

Using a LIVE/DEAD assay, we observed C2C12 cell viability and could show cell proliferation on the spider silk scaffold, suggesting that nutrient and oxygen delivery in this rotating bioreactor model were sufficient. 

Using qPCR, we confirmed the changes in gene expression of the target genes desmin, MYF5 and MEF2D, which were utilized to conclude whether the cultured myofibroblast cells did in fact undergo differentiation into active cells. Increased expressions of the differentiation markers detected in the rotating bioreactor setup indicate that dynamic conditions upheld an increased differentiation level compared to static conditions. Again, we could speculate that the increase in differentiation of the myofibroblasts could be a result of increased oxygen and nutrient supply. Similarly, the Western blot demonstrated the synthesis of α-SMA in the protein fractions, further supporting an active biological function of the smooth-muscle cells.

In the present study, we have employed murine cells to evaluate the feasibility of spider silk scaffold cell seeding in a dynamic bioreactor setup. Indeed, non-human tissues have long been implemented within the field of vascular tissue engineering. Future in vivo studies could focus on incorporating human-derived autologous cells, which may help avert possible immunoreactions to xenogeneic cell molecules. Additionally, recent innovations in stem cell technology, particularly CRISPR-mediated genome editing, have provided an encouraging instrument for genome sequence editing and have proven helpful in stem cell engineering. Niu et al. have already successfully demonstrated the feasibility of inactivating endogenous retroviruses in an immortalized pig cell line [35], sparking hopes of overcoming challenges in xenotransplantation. Additionally, induced pluripotent stem cells have been established as valid alternatives to non-human smooth-muscle and endothelial cells for vascular tissue engineering, with the benefit of providing the capacity of producing molecular components of the extracellular matrix, for instance elastin and collagen [36].

Future studies will have to assess how endothelial cells can be incorporated to create an anatomically equipped and functional vessel. Possible ways of implementing endothelial cells in the described construct could be the bilateral seeding of smooth-muscle cells and endothelial cells and the following formation of a tubular construct. In addition, the scaffolds may be formed into tubular structures by sheet rolling or matrix molding after myoblast seeding, and endothelial cells may be manually injected within the dynamic bioreactor system into the newly formed vessel lumen.

In summary, our study offers proof of the functional growth of myofibroblast cells on a spider silk scaffold for constructions of tissue-engineered vessels. Additionally, we showed a proof-of-concept regarding the optimization of growth in a rotating bioreactor model. Our findings may support the prospect of further developments in vessel tissue engineering with a simpler bioreactor setup for various clinical applications and could provide an additional basis to warrant clinical trials. 

## 5. Conclusions

We demonstrated that dynamic culturing of C2C12 cells on spider silk scaffolds in a rotating bioreactor optimizes cell viability and morphology and substantially increases the distinctive markers for active smooth-muscle cells. In contrast to pulsatile bioreactors, the rotating bioreactor allowed for a simplified experimental setup to generate viable smooth-muscle cells for application in the tissue engineering of vessels. 

## Figures and Tables

**Table 1:** Primers used for the analysis of gene expression.

Primer	Forward′➔3′	Reverse 5′➔3′	NCBI Accession No.
MYF5	GCTTTCGAGACGCTCAAGAG	GGACAAGCAATCCAAGCTG	NM_008656
MEF2D	CAAGCTGTTCCAGTATGCCAG	AAGGGATGATGTCACCAGGG	NM_001310587
Desmin	AGAAGCCGATCCAGGCAAAA	AAGGGATGATGTCACCAGGG	NM_010043
B2M	ATGAGTATGCCTGCCGTGTGGA	GGCATCTTGCAAACCTCCATG	NM_004048
RPL37	GCGTGATATAGCGGAAGTGC	ACTTCTGAAGGTGGTAGGCC	NM_009097
TBP	GCAGTGCCCAGCATCACTAT	CACAAGGCCTTCCAGCCTTA	NM_013684.3

## Data Availability

The data presented in this study are available on request from the corresponding author.
